# Identification of conserved miRNA molecules in einkorn wheat (Triticum monococcum subsp. monococcum) by using small RNA sequencing analysis

**DOI:** 10.3906/biy-1802-3

**Published:** 2018-12-10

**Authors:** Ercan Selçuk ÜNLÜ, Sara BATAW, Didem ASLAN ŞEN, Yunus ŞAHİN, Nusret ZENCİRCİ

**Affiliations:** 1 Department of Biochemistry, School of Medicine, Altınbaş University , İstanbul , Turkey; 2 Department of Biology, Faculty of Arts and Science, Abant İzzet Baysal University , Bolu , Turkey; 3 Department of Chemistry, Faculty of Arts and Science, Abant İzzet Baysal University , Bolu , Turkey

**Keywords:** microRNA, wheat, Perl, Mfold, small RNAs

## Abstract

Triticum monococcum subsp. monococcum as a first cultivated diploid wheat species possesses desirable agronomic and quality characteristics. Drought and salinity are the most dramatic environmental stress factors that have serious impact on yield and quality of crops; however, plants can use alternative defense mechanisms against these stresses. The posttranscriptional alteration of gene expression by microRNAs (miRNAs) is one of the most conserved mechanisms. In plant species including wheat genomes, miRNAs have been implicated in the management of salt and drought stress; however, studies on einkorn wheat (Triticum monococcum subsp. monococcum) are not yet available. In this study, we aimed to identify conserved miRNAs in einkorn wheat using next generation sequencing technology and bioinformatics analysis. In order to include a larger set of miRNAs, small RNA molecules from pooled plant samples grown under normal, drought, and salinity conditions were used for the library preparation and sequence analysis. After bioinformatics analysis, we identified 167 putative mature miRNA sequences belonging to 140 distinct miRNA families. We also presented a comparative analysis to propose that miRNAs and their target genes were involved in salt and drought stress control in addition to a comprehensive analysis of the scanned target genes in the T. aestivum genome.

## 1. Introduction

Wheat is one of the leading global crops, with an annual production of over 615.8 million metric tons. The level of its polyploidy is an important criterion for the classification of wheat species. Even though the time and the location are not clear, wild diploid wheat was spontaneously evolved from its close relative, Triticum boeticum Boiss. Wild emmer emerged as a tetraploid wheat form and rehybridization of this form over time with a diploid close relative resulted in the rise of spelt-like hexaploid wheat. Due to the influence of human practices, wild diploid and tetraploid plants have undergone genetic selection for their useful agronomic traits. This evolution process resulted in the cultivation of diploid (e.g., einkorn) and tetraploid (e.g., emmer) wheat forms. Wheat is classified under the genus Triticum of Triticeae (Briggle, 1963) , and several species have been characterized with diverse morphological and genetic variations (Curwen-McAdams et al., 2016) .

T. monococcum subsp. monococcum (einkorn wheat) is a diploid wheat derived from T. boeoticum (wild einkorn wheat). It is capable of growing in adverse environmental conditions. It has a high nutritional value and gives acceptable yield on poor soils. Cultivation of einkorn wheat dates back to early times of the first agricultural activities. Since then, it has been cultivated in some provinces of Turkey (Karagöz and Zencirci, 2005), the Balkan countries, and Morocco (Serpen et al., 2008) .

Small RNA molecules are noncoding RNA elements with a diverse group of functions. Several classes of small RNAs (e.g., miRNAs, siRNAs, and piRNAs) have been described (Peters and Meister, 2007) . MicroRNA (miRNA) molecules are short and single-stranded noncoding RNA molecules acting as posttranscriptional control elements in animals, plants, and fungi (Bartel, 2004; Carthew and Sontheimer, 2009) . Biosynthesis of mature miRNA molecules requires a chain of biochemical reactions starting with the transcription process carried out by Pol II or Pol III enzymes, which yields the primary miRNA (Pri-miRNA) molecules. Pri-miRNA is folded into a stem-loop structure that is then systematically digested to produce approximately 21–23-nt-length mature miRNA molecules (Ritchie et al., 2007). Studies on plants have shown that miRNA molecules have crucial roles in growth, development, and stress resistance processes (Jones-Rhoades et al., 2006; Zhang et al., 2006; Budak and Akpinar, 2015) .

miRNAs have been discovered from various organisms, and, to date, a total of 1269 plant miRNAs have been identified and deposited in MirBase (GritfihsJones et al., 2008). There are more than a hundred entries available for popular plant species, and for some important plants the unique reads are listed as 600 for Oryza sativa, 573 for Glycine max, 508 for Arabidopsis thaliana, and 158 for Zea mays as of 1 March 2018. The numbers drop dramatically for plant species with limited or unknown genome sequences, such as 118 for Triticum aestivum, 116 for Hordeum vulgare, 102 from Solanum lycopersicum, 18 from Vigna unguiculata, 16 from Saccharum oficinarum , and 12 from Phaseolus vulgaris (http://www.mirbase.org).

In a recent comprehensive study, 88 miRNA reads for T. monococcum subsp. monococcum were predicted by the homology-based analysis of putative miRNA sequences from the transcriptome assemblies in the NCBI database (Alptekin and Budak, 2016) . The numbers of miRNAs identified from other plant species suggest that more miRNA molecules are yet to be identified from T. monococcum subsp. monococcum. In particular, we hypothesize that identification of miRNAs involved in stress regulation requires alternative strategies since most of those miRNAs are expressed during stress conditions. In the present study, we pooled the samples of T. monococcum subsp. monococcum tissues grown under normal conditions and from those subjected to salt and drought stress to increase the number of identified miRNAs, and analyzed the sequences of extracted small RNA molecules to identify the expressed miRNA sequences.

## 2. Materials and methods

### 2.1. Sampling of T. monococcum subsp. monococcum cultures

Einkorn seeds belonging to six different wheat populations were surface-sterilized using 70% ethanol and 30% sodium hypochlorite. Seeds were germinated on half-strength MS solution. The cultures were incubated for 10 days in a growth chamber under controlled conditions at 24 ± 2 °C with a 16-h light and 8-h dark photoperiod before the stress treatment.

After 10 days, the plant samples were grown under control (no treatment), salt stress (100 mM NaCl), and drought stress (0.3 MPa PEG-600) conditions in a growth chamber under the same conditions as defined above (Mahmood et al., 2002) . Leaf and root samples from control and treated plants were harvested after 0, 3, 9, 12, and 24 h of the stress application and immediately frozen in liquid nitrogen. Approximately 4.0-g samples of the pooled wheat tissues from all the treated and control wheat samples were submitted to Source BioScience Plc (Nottingham, UK) for RNA isolation, small RNA library preparation, and sequencing using the Illumina MiSeq next generation sequencing platform.

### 2.2. Small RNA isolation

Small RNA molecules (<200 nt) were extracted from the pooled samples using a mirVana miRNA Isolation Kit according to the manufacturer’s instructions (Life Technologies). The sample was quantified using an Agilent 2100 Bioanalyzer to ensure that the quantity and quality of the submitted material met the specified criteria before progressing through the library preparation.

### 2.3. Small RNA library construction and sequencing

The library was prepared using a TruSeq Small RNA Sample Preparation Kit. The 3` and 5` adapters were ligated to each end of the RNA molecule, and a reverse transcription reaction was used to create single stranded cDNA. Then cDNA fragments having adapter molecules on both ends underwent 11 cycles of PCR to amplify the amount of prepared material. The resulting library (18.35 nM) was validated with the Agilent 2100 Bioanalyzer. The library was then loaded onto an Illumina MiSeq Flow Cell at a concentration of 8 pM, and the samples were then sequenced using 50-bp paired-end runs.

### 2.4. Computational sequence analysis

#### 2.4.1. Trimming and collapsing sequences

Before starting blast analysis, the data were cleaned of redundant sequences. First, the sequences were adapted and quality-trimmed using Skewer (version 0.1.12) (Jiang et al., 2014). The trimming parameters were adjusted for the small RNA input. The first processing step of merging identical reads and saving their occurrences (collapsing) was performed in order to provide data in the least redundant way possible and to speed up the classification process.

#### 2.4.2. Profiling of small RNAs

For the general classification of available small RNA molecules, the collapsed sequence data were mapped to the A. thaliana genome as a reference from Ensembl (TAIR10) (Kersey et al., 2016) using Bowtie (Langmead et al., 2009) and filtered for known RNA elements. The detailed analysis of the small RNA library sequences received from the Illumina MiSeq platform was analyzed using Perl codes designed by our group as described by Ünlü et al. (2015). Basically, the blast code was generated to analyze small RNA sequences compared with the database generated using formatdb (Altschul et al., 1990) from a total of 30,424 known mature miRNA sequences belonging to 203 different species available at miRBase (Kozomara and Gritfihs-Jones, 2011) . Given the short sequences, the code selected sequences having more than a 90% identity to reduce the risk of false positives.

#### 2.4.3. Prediction of secondary structures

For the secondary structure prediction studies, a previously designed Perl code was used to identify precursor miRNA sequences (Ünlü et al., 2015). The code searches for 100% matches for putative mature miRNA sequences in T. monococcum subsp. monococcum chromosomal scaffold sequences (downloaded from NCBI). When a match is located, the sequence is extracted along with 80 nucleotides upstream and downstream of the located miRNA. Then a prediction of the secondary structures of the extracted miRNA precursor sequences was carried out using the RNA Folding Form application (http://mfold.rna.albany. edu) (Zuker, 2003) with the default software settings. The structural output files in the ct file format were uploaded to the Mfold server using the Structure Display and Free Energy Determination application to display the structure (Mathews et al., 1999) .

#### 2.4.4. Bioinformatics analysis for the characterization of putative miRNA target genes

To identify the target genes for the predicted miRNAs in this study, the complementary sequence matches were screened in A. thaliana and T. aestivum genomes using the psRNATarget tool (Dai and Zhao, 2011) . We set the parameter to default values except for the maximum expectation being set to 3.0, the length for complementarity scoring (hspsize) being set to  18, and the number of top target genes for each small RNA being set to 50. For the categorization of target genes and the extents of their involvement in stress control, we downloaded the list of A. thaliana genes from the TAIR database (http://www.arabidopsis.org) and filtered those annotated as "response to stress" under the GO Slim functional category. Then we compared the list of predicted target genes in terms of whether they belonged to any of the filtered 519 stress-related genes.

For a detailed annotation analysis of miRNA target genes in T. aestivum, we downloaded the annotation file (Version 2.2) (Mayer et al., 2014) from the Joint Genome Institute portal that reported the protein-coding gene sequences in T. aestivum. We extracted the information for the genes showing the target fingerprints against the identified miRNA sequences. Using simple Perl codes (available from https:// github.com/esunlu/go_cluster_analysis), we clustered the GO annotations for the terms "biological process", "molecular function", and "cellular component" and analyzed the data to obtain a detailed functional categorization.

## 3. Results

### 3.1. Prediction of small RNAs

**Figure 1:**
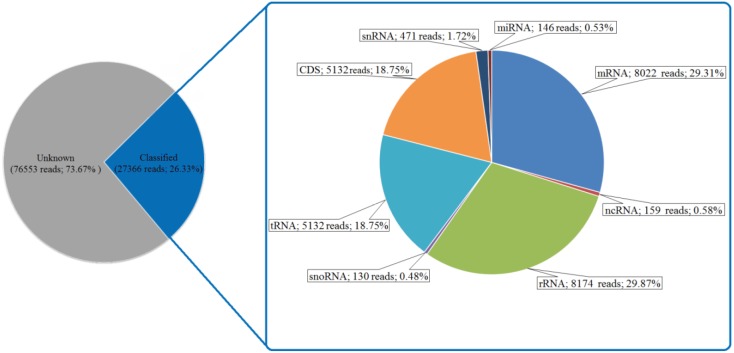
Distribution of small RNA molecules in T. monococcum subsp. monococcum sequence data.

An average of 4.0 g pooled wheat tissues were processed to prepare the small RNA sequencing library. After sequencing, 15,139,448 raw reads were obtained. The reads were processed by trimming adapter sequences, quality filtering, and merging identical reads, yielding 751,647 identical small RNA sequences. The results were further filtered against several databases of known elements in A. thaliana in which the largest family of small RNAs was identified including miRNAs, CDS, mRNAs, tRNAs, snoRNAs, ncRNAs, snRNAs, and rRNAs (Figure 1).

**Figure 2:**
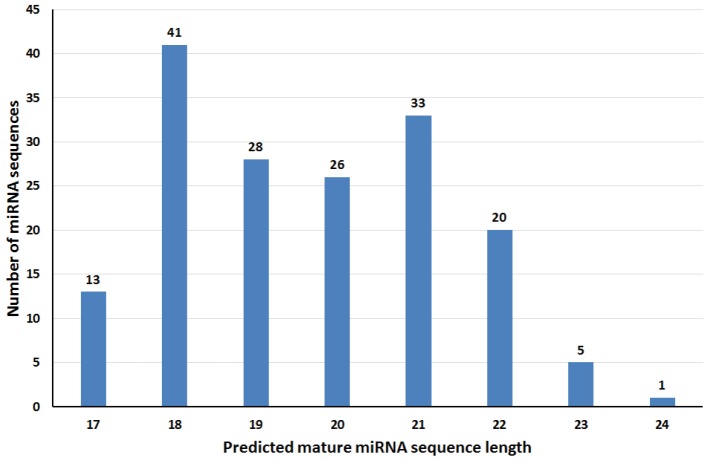
Length distribution of predicted miRNA molecules.

From the miRBase database, 30,434 known mature miRNA sequences belonging to 203 different species were obtained and formatted for blast analysis. To reduce the numbers of false positives, we set the parameters to >90% identity and >0.0001 E-value in our blast code. The analysis identified 167 putative mature miRNA sequences belonging to 140 miRNA families (shown in S1 Table). When the sequence lengths were compared, the most abundant read length was 18 nucleotides (24.55%), followed by 21 nucleotides (19.76%) and 19 nucleotides (16.77%) among the total identified miRNAs (Figure 2).

**Figure 3:**
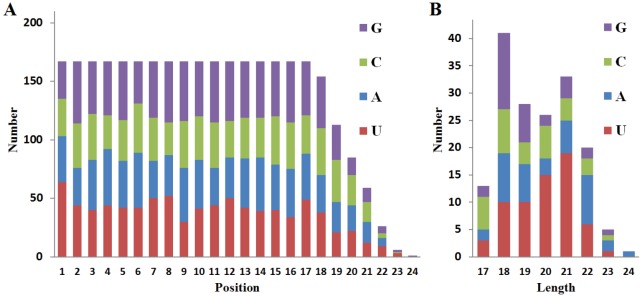
Nucleotide distribution analysis. Analysis for the first nucleotide (A) and positional (B) biases.

The base distribution analysis at each position of the identified miRNA sequences revealed that uracil and guanine were the most abundant in the first and second positions with 64 and 53 of the sequences, respectively (Figure 3A). In addition, when the base distribution was analyzed against the length of miRNAs, a dominant bias towards uracil (U) at the first nucleotide was found especially for miRNAs with a length of 19–21 nt (Figure 3B).

### 3.1.1. Validation of T. monococcum subsp. monococcum miRNAs by secondary structure prediction

Since there are no available sequence data for T. monococcum subsp. monococcum chromosomes, the Triticum urartu chromosomal scaffold sequence data were downloaded from NCBI and used as reference to extract the precursor miRNA sequences. Using the encoded Perl script, 1,455,436 scaffold sequences, with sequence lengths ranging from 50 to 82,078 nucleotides, were searched for 100% positive matches. The extracted sequence frame corresponds to 80 nucleotides upstream of the start of the matching mature miRNA and 80 nucleotides downstream of miRNA.

We were able to extract 111 precursors to be analyzed for characteristic secondary structure folding. Mfold software was used to analyze secondary structures of the extracted pre-miRNA sequences. The default parameters were used to analyze secondary structures of the selected sequences. Seventy-seven of the sequences showed a stem-loop structure that is characterized for pre-miRNA sequences (see S2 Table). It is obvious that completing the assembly of T. monococcum subsp. monococcum of T. urartu chromosomal sequences will enhance the potential of the bioinformatics analysis for the Triticum species. The failure to predict of the secondary structure for the remaining 34 T. monococcum subsp. monococcum miRNAs was likely to have been due to the incomplete and fragmented nature of the T. urartu scaffold sequences used for the analysis.

### 3.2. Characterization of putative miRNA targets by bioinformatics prediction

Target prediction is an important step to characterize miRNA function. We compared the predicted miRNAs to those verified by experimental analysis in other plants under drought and salt stress conditions (Table 1). In this study, 23 salt stress and 24 drought stress-related miRNAs were identified for T. monococcum subsp. monococcum.

**Table 1:** A comparison of the miRNA families identified for T. monococcum subsp. monococcum in terms of their responsiveness to drought and salt stress in different plant species.

miR	Status of expressional verification									
	T. aestivum		H. vulgare		A. thaliana		Z. mays		O. sativa	
	Salt^a^	Drought^f^	Salt^b^	Drought^g^	Salt^c^	Drought^c^	Salt^d^	Drought^i^	Salt^e^	Drought^e^
miR156			√	√	√		√	√	√	√
miR157						√				
miR159		√		√	√		√	√	√	
miR160	√	√					√	√		√
miR164	√		√				√			
miR165					√					
miR166		√	√	√			√	√		√
miR167					√	√	√	√		√
miR168				√	√	√	√	√		√
miR169	√	√	√	√	√			√		
miR171			√		√	√	√			√
miR172	√	√		√						√
miR319					√		√		√	√
miR393				√	√	√				√
miR394					√				√	
miR395	√	√								√
miR396	√				√	√		√		√
miR397										√
miR398								√		
miR408			√			√		√		√
miR444	√			√						
miR528									√	
miR529	√									√
miR530									√	
miR535	√									
miR845										√
miR894								√		
miR1125										√
miR5048				√						
miR5049	√	√		√						

a (Eren et al., 2015), b (Deng et al., 2015), c (Liu et al., 2008), d (Ding et al., 2009), e (Barrera-Figueroa et al., 2012; Zhou et al., 2010), f (Akdogan et al., 2016), g (Hackenberg et al., 2015), i (Wei et al., 2009)

**Figure 4:**
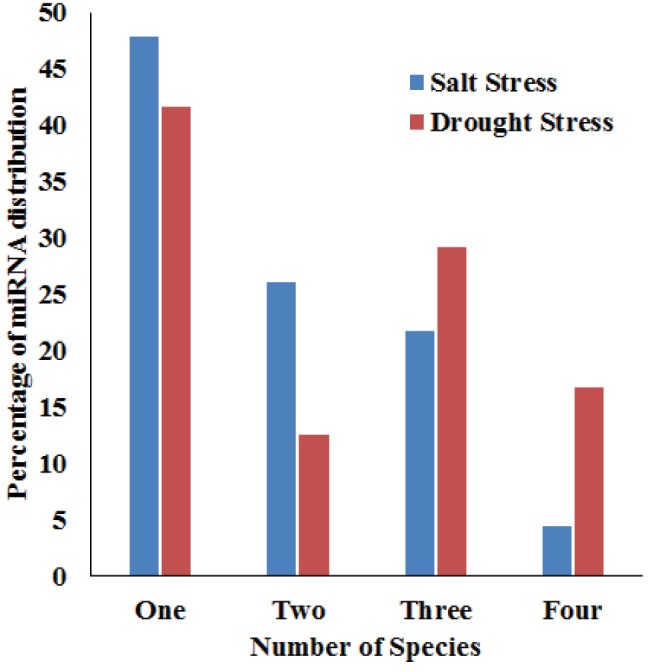
Comparison for number of salt and drought stress related miRNAs among species. Data summarize the conservation level of stress related miRNA among other plant miRNAs that are known to be associated with stress tolerance functions.

A comparison of the data among different plants suggests that most of the stress-related miRNAs are common across the compared species. According to the literature data as listed in Table 1, co-expression of 17 identified miRNAs was associated with both salt and drought stress conditions, experimentally (qPCR, northern blot, microarray, etc.). In addition, more than half of the salt and drought stress-related miRNAs were conserved in at least two different species (Figure 4). None of the miRNAs controlling either condition was conserved in all five of the analyzed species.

We used the psRNATarget tool to scan possible target genes for sequences of the predicted miRNA families presented in Table 1. For the salt response-related miRNA targets, of the 1435 genes that were identified, 78.33% were proposed to be controlled by cleaving the corresponding transcript. For the drought stress-related miRNA target genes, it is proposed that 79.01% of 1548 genes were controlled by cleaving the corresponding transcript. To enrich our data in terms of the stress responsive gene targets, we compared the identified target genes with A. thaliana stress-related genes. In this study, the 22 miRNA families that were identified revealed that 30 target genes were directly related to stress in A. thaliana. A list of the names of the miRNAs and the predicted targets is presented in Table 2. To validate whether those A. thaliana target genes are conserved in wheat, we carried out a blast analysis for the target gene products containing T. urartu proteins. All the proteins are verified in the T. urartu genome but five of them are yet to be functionally characterized (Table 2).

**Table 2:** Summary of the target prediction of stress related miRNAs.

miR family	miR name	Drought	Salt	GenBank (T. urartu)	Protein product (T. urartu)
miR156	miR156-3p	√	√	EMS63385.1	Argonaute 1B
miR159			miR159-3p	√	√	EMS55264.1	IAA-amino acid hydrolase ILR1-like 5
miR159-3p	√	√	EMS66412.1	IAA-amino acid hydrolase ILR1-like 3
miR159-5p	√	√	EMS59656.1	Acetyl-CoA carboxylase
miR165	miR165	x	√	EMS60006.1	3-ketoacyl-CoA synthase 6
miR166		miR166	√	√	EMS47855.1	Putative glutathione S-transferase
miR166-5p	√	√	EMS67450.1	Zinc finger CCCH domain-containing protein 45
miR168						miR168-5p	√	√	EMS63385.1	Protein argonaute 1B
miR168	√	√	EMS63385.1	Protein argonaute 1B
miR168-5p	√	√	EMS48655.1	Proline-rich receptor-like protein kinase PERK13
miR168-5p	√	√	EMS55864.1	Mitogen-activated protein kinase 17
miR168	√	√	EMS55864.1	Mitogen-activated protein kinase 17
miR168-5p	√	√	EMS62275.1	Receptor-like protein kinase
miR169		miR169	√	√	EMS46116.1	D repeat and FYVE domain-containing protein 3
miR169	√	√	EMS61213.1	Protein TIFY 6B
miR172		miR172	√	√	EMS66886.1	Trihelix transcription factor GT-2
miR172-5p	√	√	EMS50683.1	Cell division cycle 5-like protein
miR393	miR393	√	√	EMS56796.1	Hypothetical protein
miR395		miR395-5p	√	√	EMS53304.1	Hypothetical protein
miR395-5p	√	√	EMS68547.1	Alpha-glucan water dikinase, chloroplastic
miR396	miR396-3p	√	√	EMS61897.1	ATP-dependent DNA helicase MPH1
miR398		miR398-3p	√	√	EMS67509.1	Copper chaperone for superoxide dismutase
miR398-3p	√	√	EMS45437.1	Pectinesterase
miR399		miR399	√	√	EMS47483.1	Disease resistance protein RGA2
miR399	√	√	EMS48356.1	Hypothetical protein
miR408	miR408	√	√	EMS53029.1	DNA polymerase epsilon catalytic subunit A
miR444	miR444	√	√	EMS60248.1	Hypothetical protein
miR529	miR529	√	√	EMS53316.1	Transcriptional corepressor SEUSS
miR845-5p	miR845-5p	√	x	EMS63076.1	Hypothetical protein
miR5049	miR5049	√	√	EMS46913.1	Pyruvate decarboxylase isozyme 2

**Figure 5:**
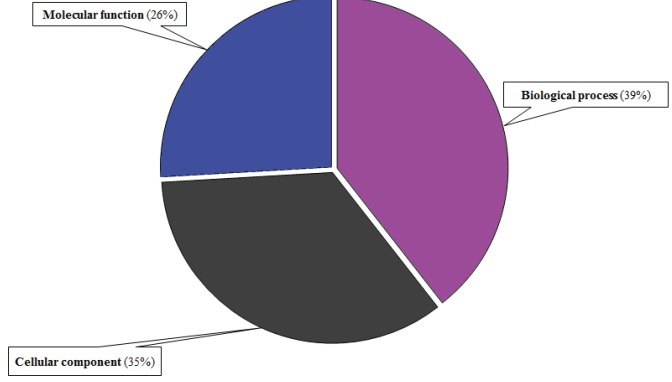
General GO term distributions for miRNAs target genes in the T. aestivum genome.

**Figure 6:**
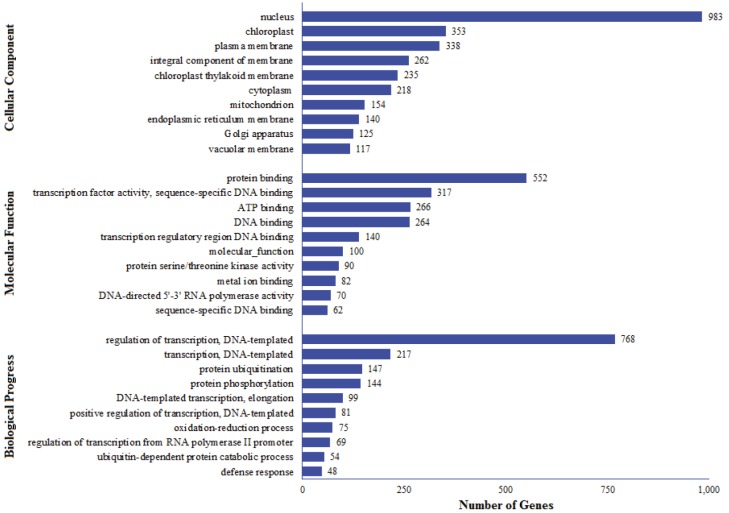
Summarized GO classification of miRNAs target genes in the T. aestivum genome. The representation of the number of genes was limited to ten functional classes showing the highest number of genes for each GO term.

We also scanned possible target genes for sequences of the predicted miRNA families in the available T. aestivum genome in order to present a more comprehensive putative target list. Screening an EMBL-based reference genome sequence revealed that 113 of the miRNA sequences statistically significantly matched the T. aestivum target genes, and 92.90% of 1085 genes were likely to be controlled by cleaving the corresponding transcript. We extracted the detailed annotation information for the target genes and clustered GO annotations under the terms "biological process", "molecular function", and "cellular component" for 908 putative target genes. We were able to retrieve 14,336 GO term matches, of which 39% were clustered for biological process, followed by 35% for cellular component, and 26% for molecular function clusters (Figure 5). A summary of the functional distribution of the matching GO terms is presented in Figure 6.

## 4. Discussion

Across the globe, wheat is one of the most demanded crops. In the present study, we aimed to fill an information gap regarding miRNA data for T. monococcum subsp. monococcum. We carried out a small RNA sequencing analysis to elucidate the miRNA sequences. To increase the number of identified miRNAs, we pooled samples of plants grown under normal, salt, and drought stress conditions. By adopting comparative genomics approaches, we successfully identified 140 distinct miRNA families covering 167 miRNA sequences.

The general sequence profiles of the identified miRNA molecules were similar to those proposed for miRNA characterization studies. Both the first nucleotide bias and the position nucleotide bias observations fit the previously described characteristics of the miRNAs (Lau et al., 2001; Ge et al., 2013) . Due to a lack of chromosomal sequence information, we were not able to analyze the secondary structures for all the identified miRNA sequences; however, we did successfully display the structure models for 77 of the 111 analyzed pre-miRNAs extracted from T. urartu chromosomal scaffold data.

We also carried out a bioinformatics analysis for the characterization of the identified miRNA sequences regarding their potential involvement in salt and drought stress regulation. We characterized 23 miRNAs as potential regulators for salt stress and 24 miRNAs as potential regulators for drought stress in T. monococcum subsp. monococcum. When we analyzed the putative targets for those miRNA sequences, our results showed that 20 target genes and their corresponding miRNAs were identical when compared with the lists for drought and salt stress, except for miR148 and mir845. This suggests that both salt and drought stress are under a common master regulator that controls both conditions, and this fits with a previously proposed model (Deng et al., 2015) . It is likely that the AGO1 gene (predicted as a target for miR168 family) acts as a master regulator for both drought and salt responsive target genes in T. monococcum subsp. monococcum as previously suggested for A. thaliana (Vaucheret et al., 2009). In addition, it was previously shown that this interaction is necessary for a salt stress response in barley, and it is directly related to the miR168 levels under salt stress conditions (Deng et al., 2015) . In fact, there is a conserved nature for regulation of miRNA machinery especially for stress response (Datta and Paul, 2015) . Thus, proposing the involvement of AGO1 in miRNA regulation during stress response would not be misestimating assumption.

In this study, we also carried out a target analysis using the T. aestivum genome as a reference. The GO annotation analysis for putative target genes affiliated with the identified miRNAs has a role in protein interactions and the regulation of mRNA levels. Data suggest that the identified miRNAs are involved in transcriptional and posttranscriptional regulatory control. The miRNA/target gene data presented in this study can be used as a reference for comprehensive functional genomics studies.

In conclusion, this study provides a large amount of putative miRNA sequence information for einkorn wheat. As a follow up, the expression profiles of proposed miRNAs and their potential targets under diverse stress conditions should be evaluated in a comprehensive study. In addition, the T. monococcum subsp. monococcum genome assembly is yet to be completed, and this is the major drawback for detailed molecular and bioinformatics studies on einkorn. Completing the assembly of at least one species among wheat can escalate further molecular studies.

## Acknowledgment

This study was supported by Abant İzzet Baysal University BAP grant, Grant No: 2015.03.01.880.

## Supplementary Material

Sequence information for the identified miRNAs.

Predicted secondary structures for putative miRNA sequences identified in T. monococcum subsp. monococcum.
