# Supplementary material for: Identification of conserved miRNA molecules in einkorn wheat (Triticum monococcum subsp. monococcum) by using small RNA sequencing analysis
Source: Turk J Biol. 2018 Dec 10;42(6):527–36. doi: 10.3906/biy-1802-3 (PMC6451844; doi:10.3906/biy-1802-3)
Supplement: Sequence information for the identified miRNAs. [file turkjbio-42-527-s001.pdf]

SUPPLEMENTARY DATA

S1 Table. Sequence information for the identified miRNAs.

Predicted miRNA family name	Predicted miRNA name	Sequence length	Copy number	Sequence
miR22	tmo-miR22	22	1	AAGCUGCCAGUUGAAGAACUGU
miR30	tmo-miR30-5p	20	1	UGUAAACAUCCUCGACUGGA
miR99	tmo-miR99	21	1	CACCCGUAGAACCGACCUUGC
miR156	tmo-miR156	20	5	UGACAGAAGAGAGUGAGCAU
	tmo-miR156-3p	21	16	GCUCACCCUCUCUCUGUCAGC
	tmo-miR156-5p	20	1	UGACAGAAGAGAGCGAGCAC
miR157	tmo-miR157	21	1	UUGACAGAAGAUAGAGAGCAC
miR159	tmo-miR159	21	1	UUUGGAUUGAAGGGAGCUCUG
	tmo-miR159-3p	18	1	UUUGCAUGACCGAGGAGC
	tmo-miR159-5p	21	1	AGCUGCUUGUUCAUGGUUCCC
miR160	tmo-miR160	21	1	UGCCUGGCUCCCUGAAUGCCA
miR164	tmo-miR164	21	7	UGGAGAAGCAGGGCACGUGCA
miR165	tmo-miR165	21	1	UCGGACCAGGCUUCAUCCCCC
miR166	tmo-miR166	21	1	UCGGACCAGGCUUCAUUCCCC
	tmo-miR166-3p	17	1	UCGGACCAGGCUUCAAU
	tmo-miR166-5p	18	2	GGUUGUUGUCUGGUUCAA
miR167	tmo-miR167	22	1	UGAAGCUGCCAGAAUGAUCUGA
	tmo-miR167-3p	18	13	UCAUGCUGGAGUUUCAUC
miR168	tmo-miR168	21	2	UCGCUUGGUGCAGGUCGGGAA
	tmo-miR168-3p	18	13	CCCGCCUUGCACCAAGUG
	tmo-miR168-5p	19	1	UUGCUUGGUGCAGAUCGGG
miR169	tmo-miR169	19	1	AGCCAAGGAUGACUUGCCA
	tmo-miR169-3p	19	4	GGCAGUCUCCUUGGCUAGC
miR171	tmo-miR171	21	3	UUGAGCCGUGCCAAUAUCACG
	tmo-miR171-3p	20	2	UUGAGCCGUGCCAAUAUCAC
	tmo-miR171-5p	19	1	UGGUAUUGUUUCGGCUCAU
miR172	tmo-miR172	19	2	GAAUCUUGAUGAUGCUGCA
	tmo-miR172-5p	21	1	GCAGCACCACCAAGAUUCACA
miR181	tmo-miR181-5p	22	1	AACAUUCAACGCUGUCGGUGAG
miR182	tmo-miR182	18	1	GUGGCACUAGUGGAAUUC
miR319	tmo-miR319	19	2	UUGGACUGAAGGGAGCUCC
	tmo-miR319-3p	21	46	CUUGGACUGAAGGGUGCUCCC
	tmo-miR319-5p	20	3	AGAGCGUCCUUCAGUCCACU
miR390	tmo-miR390	21	1	AAGCUCAGGAGGGAUAGCGCC
	tmo-miR390-3p	19	2	GCUAUCUAUCCUGAGCUCC
miR393	tmo-miR393	21	46	UCCAAAGGGAUCGCAUUGAUC
	tmo-miR393-3p	22	1	GAUCAGUGCAAUCCCUCUGGAA
miR394	tmo-miR394	17	5	UUGGCAUUCUGUCCACC
	tmo-miR394-3p	18	1	GUGGGCAUACUGCCAAUG
miR3944	tmo-miR3944-3p	18	1	ACCUUCGGGCUGGCCUGC

S1 Table. (Continued).

miR395	tmo-miR395	21	1	UGAAGUGUUUGGGGGAACUCC
	tmo-miR395-5p	18	1	UGAAGUGUUUGAGGGAAC
miR396	tmo-miR396	20	1	UCCACAGGCUUUCUUGAACU
	tmo-miR396-3p	21	4	GGUCAAGAAAGCUGUGGGAAG
miR397	tmo-miR397	20	12	UUGAGUGCAGCGUUGAUGAA
miR398	tmo-miR398	20	1	UGUGUUCUCAGGUCACCCCU
	tmo-miR398-3p	20	1	GUGUUCUCAGGUCGCCCCUG
miR399	tmo-miR399	21	3	UGCCAAAGGAGAAUUGCCCUG
miR408	tmo-miR408	21	1	UGCACUGCCUCUUCCCUGGCU
miR414	tmo-miR414	17	1	GAGGAUGAUGAGGAUGA
miR444	tmo-miR444	19	1	UGCAGUUGCUGUCUCAAGC
	tmo-miR444-3p	21	8	UGCAGUUGCUGCCUCAAGCUU
miR456	tmo-miR456-5p	18	1	UGCACUGCCUUCAGAGUG
miR466	tmo-miR466-5p	19	1	AACACACACACACACACAC
miR479	tmo-miR479	21	1	UGAGCCGAACCAAUAUCACUC
miR529	tmo-miR529	21	1	AGAAGAGAGAGAGUACAGCCC
	tmo-miR529-3p	18	1	GCUGUACCCUCUCUCUUC
miR530	tmo-miR530	20	1	CUGCAUUUGCACCUGCACCU
miR535	tmo-miR535	22	1	UGACAACGAGAGAGGGCACGCG
miR619	tmo-miR619-5p	22	1	GCCUCGGCCUCUCAAAGUGCUG
miR650	tmo-miR650	21	1	CCAUGGUGGAGAUGUCCUGAG
miR706	tmo-miR706	22	1	CCAGGGCUAUACAGAGAAACAC
miR716	tmo-miR716	19	1	CGAGCCCGGGCGGAGCGGC
miR767	tmo-miR767-5p	23	1	UGCACCAUGGUUGUCUGAGCAUG
miR827	tmo-miR827	21	1	UUAGAUGACCAUCAGCAAACA
	tmo-miR827-5p	22	1	UCUGAACUUGUUUUGCUGGUUG
miR845	tmo-miR845-5p	19	1	ACCUUGCUCUGAUACCAAU
miR894	tmo-miR894	20	2	UUCGUUUCACGUCGGGUUCA
miR928	tmo-miR928	17	1	GUGGCUGUGGAAGCUGG
miR1117	tmo-miR1117	19	1	UUAGUACCGGUUCGUGGCA
miR1120	tmo-miR1120	18	1	AUUUUUAUAUUAUGAGAC
miR11214	tmo-miR11214	20	1	UAGUGAUCUAAACGCUCUUA
miR1122	tmo-miR1122	19	1	GUCUAGAUACGGAUGUAUC
miR1125	tmo-miR1125	24	1	AAAUUUAACCAACGAGACCAACUG
miR1131	tmo-miR1131	18	1	CUUUAGUACCGGUUCGUG
miR1133	tmo-miR1133	18	1	AAGUUUUUUCGGACGGAG
miR1135	tmo-miR1135	23	1	CCGUUCGGAAUUACUUGUCGCAG
miR1136	tmo-miR1136	22	2	ACUUGUCGCAGGUAUGGAUAUA
miR1137	tmo-miR1137	18	1	AGUUAGUACAAAGUUGAG
miR1139	tmo-miR1139	22	1	AUGUUACUAGUGUAUGUUACUC
miR1207	tmo-miR1207-5p	18	1	GGGGCAGGGAGGCAGGGA
miR1273	tmo-miR1273	22	1	AAUGAUUCGAUCUCGACUCACU
	tmo-miR1273-3p	18	1	GUCCUGCUCUGUCACCCA

S1 Table. (Continued).

miR1285	tmo-miR1285	20	1	CAGAGGUUGCAGUGAGUGGA
miR1432	tmo-miR1432-5p	20	2	UCAGGAGAGAUGACACCGAC
miR1436	tmo-miR1436	19	1	AUUAUGGGACGGAGGGAGU
miR1520	tmo-miR1520	18	1	CCCAUCACGUGUCAUGUU
miR1584	tmo-miR1584	18	1	AGGAUCAAGGGAAUCGGG
miR1878	tmo-miR1878-3p	23	2	AUUUGUAGUGUUCGGAUUGAGUU
miR2111	tmo-miR2111-5p	21	1	UAAUCUGCAUCCUGAGGUUUA
miR2120	tmo-miR2120	18	1	GAACCGGGACUAAAGAUC
miR2478	tmo-miR2478	18	1	AGAGGGCGUGGGUUCAUA
miR2525	tmo-miR2525	19	1	UUUGAUCCACUUCGCUGUC
miR2538	tmo-miR2538-5p	18	1	AUCCUCUAUUAUUUUAGU
miR2673	tmo-miR2673	18	1	CUUUUCUUCCUCUUCCUC
miR2916	tmo-miR2916	20	1	CAAGAACGAAAGUUGGGGAC
miR2919	tmo-miR2919	19	1	CCUGCCGUCGCUGUGCUUC
miR3348	tmo-miR3348	17	1	CCUCGCCGGGAGGCUCG
miR3630	tmo-miR3630-3p	17	31	AUGGGAAUCUCUCUGAU
miR3682	tmo-miR3682-5p	18	1	AGGAUAACACAGGUAGAA
miR3711	tmo-miR3711	18	3	GCCCUCCUUCUAGCGCCA
miR3885	tmo-miR3885-5p	19	1	UGCUGAGCGGCGGCCGCCG
miR3887	tmo-miR3887-3p	18	1	GGAGAGAUGGCUGUGGAA
miR4922	tmo-miR4922	18	1	UAAAUUGUAUCAUUUUUC
miR4995	tmo-miR4995	21	6	CAUAGGCAGUGGCUUGGUUAA
miR5021	tmo-miR5021	18	1	CUACAAUUUCUUCUUCUU
miR5048	tmo-miR5048	20	1	UAUAUUUGCAGGUUUUAGGU
miR5049	tmo-miR5049	23	1	AGCUGAGACACUUAUUUUGGGAC
	tmo-miR5049-3p	20	1	CAAGUAAUAUGGAUCGGAGG
miR5050	tmo-miR5050	17	1	UUUUGCUGGUUGAACGA
miR5054	tmo-miR5054	18	1	AACCACGUGGCCGUGGGU
miR5056	tmo-miR5056	21	1	UCGGGAGGAAGAACCGGUAAU
miR5059	tmo-miR5059	17	1	CGAGCCUGGGCAGCACC
miR5062	tmo-miR5062	20	2	UGAACCUUGGGGAAAAGCCG
miR5064	tmo-miR5064	20	38	UGAAUUUGUCCAUAGCAUCA
miR5067	tmo-miR5067	18	1	UUCAUAUUAGUUGUCGCU
miR5072	tmo-miR5072	19	4	UUCUGGGUUCGUUCCCCAG
miR5073	tmo-miR5073	23	1	GUUUGGUGAAUCGGAAACAAUUU
miR5076	tmo-miR5076	21	1	UCUUUUUCCUUAAAUGGGAGC
miR5079	tmo-miR5079	22	1	UAUAAUUUGGAUUUGUUAUUUU
miR5082	tmo-miR5082	19	2	GCGAUGAUGGCCGCGCGGG
miR5083	tmo-miR5083	20	1	UAUUUAGUGUUGACCAAAUU
miR5084	tmo-miR5084	20	1	GUGAUCCUCUGCAGUACUGU
miR5096	tmo-miR5096	21	1	AGACAGGGUUUCACCAUGUUG
miR5106	tmo-miR5106	18	1	GGGUCUGUAGCUCAGUUG

S1 Table. (Continued).

miR5141	tmo-miR5141	17	1	CCGUCAGUCGCGUCGGG
miR5169	tmo-miR5169	18	1	UUGACCAAGUUUGUAGAA
miR5174	tmo-miR5174-3p	19	1	UUAUGGAACGGAGGGAGUA
miR5174	tmo-miR5174-5p	19	1	CAAAAACGCUGUUAUAUUA
miR5181	tmo-miR5181	19	1	AACUGCGACACUUAUUAUG
miR528	tmo-miR528-5p	21	2	UGGAAGGGGCAUGCAGAGGAG
miR5368	tmo-miR5368	18	1	GACCCGCGGGCCAAGGGA
miR5387	tmo-miR5387	18	1	CGAACCGGUGCUAAAGGA
miR5503	tmo-miR5503	22	1	AAUGCCUCUAGAAAGAUCCGAA
miR5523	tmo-miR5523	19	1	UAACUAGUAAAUAUGUUCC
miR5532	tmo-miR5532	22	1	UAUGGAAUAUAUGACAAAGGUG
miR5538	tmo-miR5538	22	1	ACUGUUGAGUAACGGCAGCAAG
miR5571	tmo-miR5571-5p	21	1	AUGUGAACCAAGCAAUUCUCA
miR5585	tmo-miR5585-3p	22	1	CCAGGCAAGGUGGCGGGCACCU
miR5658	tmo-miR5658	19	1	GAUGAGAUGAUGAUGAUGA
miR6173	tmo-miR6173	20	1	AUGGGAUUAGAGACCCCAGU
miR6177	tmo-miR6177	20	1	CCAUGGACAGAAGGCACUUA
miR6181	tmo-miR6181	22	1	UGCUCUUCAUGGACUGCGGCGC
miR6182	tmo-miR6182	21	1	GAGUGUGUGAUGGAUGGCUUU
miR6188	tmo-miR6188	19	1	GGAGGAUCGAUGAACCCGG
miR6191	tmo-miR6191	18	1	CUUAGAUUUGUCUAGAUA
miR6198	tmo-miR6198	22	2	CGGCUCUGUCUUGGAUGGUCAU
miR6199	tmo-miR6199	18	1	CCACAGAAUUCUCACAGU
miR6203	tmo-miR6203	21	1	AGGGAUUGCAGGUCUUCUUAA
miR6204	tmo-miR6204	22	1	AGAAAUGGAAAGGAGAAUAAUU
miR6214	tmo-miR6214	20	1	ACGACGACGACGAGCACGAC
miR6219	tmo-miR6219-5p	18	1	UGUAAGAACCGGGACUAA
miR6244	tmo-miR6244	19	1	CCUUGUGGUCGUGGGUUCG
miR6250	tmo-miR6250	20	1	UGCCGCCAAUCUUCUCGGGG
miR6253	tmo-miR6253	19	1	AGGAAAGUGGGCAGUUGGG
miR6300	tmo-miR6300	18	1	GUCGUUGUAGUAUAGUGG
miR6478	tmo-miR6478	20	1	CCGACCUUAGCUCAGUUGGU
miR6621	tmo-miR6621-5p	19	1	AUCUGGUACAACAGCCUGU
miR6874	tmo-miR6874-3p	18	1	UUUACCUAGUUCUGCUGU
miR6981	tmo-miR6981-5p	22	1	AGAGGAGAAGGAAGAAGCUGAA
miR7042	tmo-miR7042-3p	18	1	GUAUCAAGAGAGAAAACA
miR7116	tmo-miR7116-3p	18	1	UCCUUUUUCCUUUGCCUU
miR7398	tmo-miR7398-3p	17	1	CGUAAGAGAAGGGAGAA
miR7757	tmo-miR7757-5p	17	2	CACAAAACCUUCAGCUA
miR8155	tmo-miR8155	17	6	ACCUGGCUCUGAUACCA
miR-B6	tmo-miR-B6-3p	17	1	CGUCUCCGGCGCCGGGU
miR-I5	tmo-miR-I5-3p	18	1	GGAUGAAGAAGACGACGA
