# Supplementary material for: Identification of conserved miRNA molecules in einkorn wheat (Triticum monococcum subsp. monococcum) by using small RNA sequencing analysis
Source: Turk J Biol. 2018 Dec 10;42(6):527–36. doi: 10.3906/biy-1802-3 (PMC6451844; doi:10.3906/biy-1802-3)
Supplement: Predicted secondary structures for putative miRNA sequences identified in T. monococcum subsp. monococcum. [file turkjbio-42-527-s002.pdf]

S2 Table. Predicted secondary structures for putative miRNA sequences identified in *T. monococcum* subsp. *monococcum*.

Predicted miRNA name	Predicted secondary structure
tmo-mir-156-3p	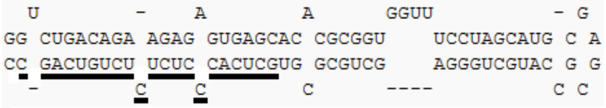
tmo-mir-156-5p	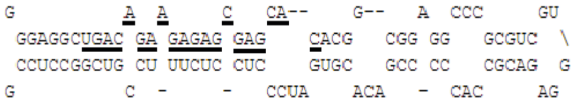
tmo-mir-159	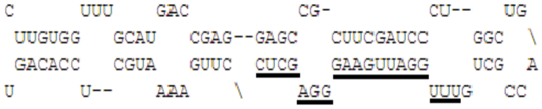
tmo-mir-159-3p	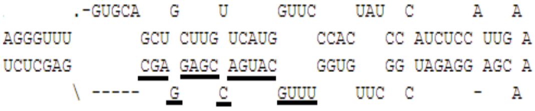
tmo-mir-159-5p	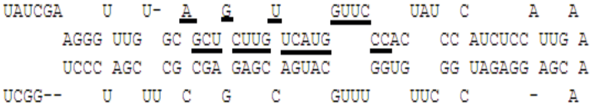
tmo-mir-160	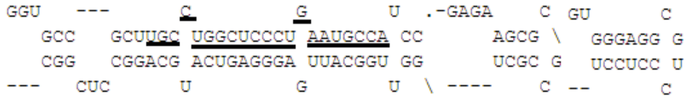
tmo-mir-164	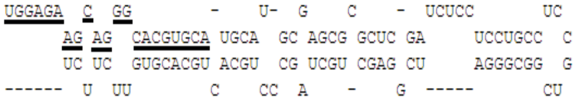
tmo-mir-166	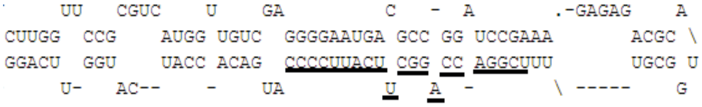
tmo-mir-166-3p	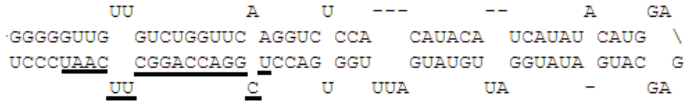
tmo-mir-166-5p	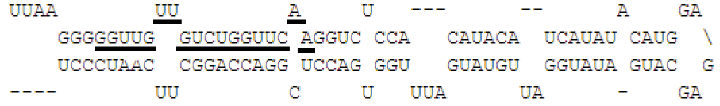
tmo-mir-167-3p	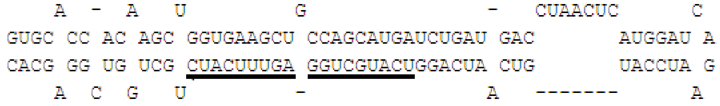
tmo-mir-169	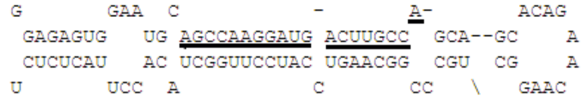
tmo-mir-169-3p	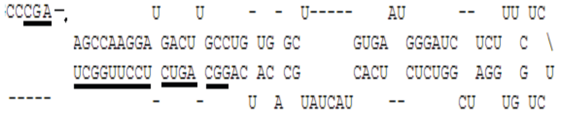

S2 Table. (Continued).

tmo-mir-171	GG U -- -- UG AG A U UG AGGA<br>--UGCGAG GAG GAA CGCG GUAUUGG CGGUUCAAUC AG GC GG CCCC \\<br>ACGCUC UUC CUU GCGC UAUAACC GCCGAGUUAG UC CG CC GGGG G<br>\ AG U UG AC GU CU C - UU AACA
tmo-mir-172	C C A GA-- - U U<br>GGUGCAGCA CA CAAGAUUC CAUCG UC CGUCG CGUAAAU \\<br>CUACGUCGU GU GUUCUAAG GUAGU AG GCAGC GUAUUUA A<br>A A A GGAC C - A
tmo-mir-172-5p	UU A C C A GA-- - U U<br>GC GGUGCAGCA CA CAAGAUUC CAUCG UC CGUCG CGUAAAU \\<br>CG CUACGUCGU GU GUUCUAAG GUAGU AG GCAGC GUAUUUA A<br>A- A A A A GGAC C - A
tmo-mir-319-3p	ACCGU--- U CU- -- U UUUCU CC<br>CGU GCUUGGA GA AGGG G UCGGGCG A<br>GUA CGAAUCU CU UCCC C AGUCCGC U<br>GAUGUAUU C CUC CC U AGCU- CA
tmo-mir-384-5p	C .-A UG AA CCA --- UCC- AU CCGUA<br>AAGGAG GCA AGC \\ GGCAG GUCA UC GUGCUUA \\<br>UUCCUC CGU UCG G CUGUC CGGU AG CACGAAU G<br>- \\ - UG CC CAC UUA UUAU AC CUUAA
tmo-mir-393	A U U CAUCCA -- GC<br>GGGGAAGC UCCAAAGGGAUCGCAU GAUCC UC UGGU GUUGAUG \\<br>CUCCUUCG AGGUUUUCCUAGCGUA CUAGG AG ACCA UAACUAC U<br>A - C CUCG-- AC UG
tmo-mir-393-3p	A U A C -- ------ U<br>GGA GC AGUGGAGGAUUCCA AGGGAU GCAUUGAUCCAUC UCU CCG A<br>CCU CG UCGCCUCUUAAGGU UCCCUA CGUGACUAGGUAG AGA GGC A<br>C U C A CU ACUCGC C
tmo-mir-397	- C AAAGG C A GCGUUGAUG - UA- C<br>GGAGGAAG AGA GC UG CAUUG GUGCA AACCG UCC CUC U<br>CCUCUUUC UCU CG GC GUAAC CACGU UUGGC AGG GAG C<br>G - ----- A A --------- G CCG C
tmo-mir-398	A CCA .-A G- UC<br>CGAUCCAGAGG GUG CUGAGAACAC AGCGC GGC C<br>GUUGGGUUUCC CAC GACUCUUGUG UCGUG CUG U<br>C UG- \\ - GA UU
tmo-mir-399	U- UGUA- --- AG UG G AGU<br>GGCAUGGU--GGCA GCA CC GGUA UG CGGC UGC U<br>UCGUACCA CCGU CGU GG CCGU GC GCCG ACG U<br>\\ CC UAAGA AAA -- GU A GUC
tmo-mir-414	UG G A ACAAAG GAA<br>GUCGU AUUU UGUCA CA G<br>CAGUA UAGG GUAGU GU A<br>-- G A AGGA-- AUU
tmo-mir-466-5p	-- - A GGA C<br>UGUGUGU UGUGUG UG UUG A<br>ACACACA ACACAC AC GAC U<br>AC C A AA- A
tmo-mir-528-5p	G- A CG U G C - UGG UGCUU<br>AGC GCAG GUGGAAGGGGCA GCA AGGAG G GCCA GAGCUU \\<br>UCG CGUC UACCUUCUCCGU CGU UCCUC C CGGU CUCGGA G<br>AG G CU C G U U --- UCUCC
tmo-mir-530	-CAUA U UU U CAG GGA AGAGAG -- AAAG A<br>CU GGU GCA GU GCAAG AGCU CCA GC UGC \\<br>GA CCA CGU CA CGUUU UCGA GGU UG ACG C<br>---- U -- C --- ACG GAUAG- AU GGAA A

S2 Table. (Continued).

tmo-mir-827-5p	U -- CU AU UAUAUCUCUAGUUCAU CU UUGUUUU C UC<br>CGCCA CU CA CG GCAG CUGAAC G UGGUUG \\<br>GUGGU GA GU GC CGUC GACUUG C ACCAAU A<br>- UC AU CU --------------- U- ------- U CU
tmo-mir-845-5p	C UAAA .-CCA GG AAAAUCACA--- A UGA<br>AUAAC ACU GGC U CAGA CAAG \\<br>UAUUG UGA CCG U GUCU GUUC G<br>A UG-- \\ --- AA AAGUUAACCAUA C CAU
tmo-mir-1117	-- UU AU GGUA<br>UC UAGUACCGGUUCGUGGC GAACC \\<br>AG AUCAUGGCCAAGCACCG CUUGG C<br>CU UU -- AAAU
tmo-mir-1120	UGAGA C C UC UC UUUUAA<br>GUACUA UC CU GU UAUAAUAUAG G<br>CAUGAU AG GA CA GUAUUAUAUU A<br>----- A U GA GA UUUAUA
tmo-mir-1122	U - CA<br>AAUUACUUGUCUUGGAUUUGUC AGAUACGGAUGUAUCUAG ACU U<br>UUAAUGAACGGAACCUAAACAG UCUAUGCCUACAUAGAUC UGA U<br>U G UU
tmo-mir-1131	---------- A CGU C A UCU<br>GGACCUUUAGU CCGGUU GGCACGAAC GGGACUAA GG C<br>UCUGGAAAUCA GGCCAA CCGUGCUUG CCCUGAUU CC A<br>UGAUUUCUAA G ACU U A CCA
tmo-mir-1133	-GAGAAAA A- C--- C CA CUAA<br>CUU UCC AAGCUUGUCCCU AAA GAUGUAUCUAACA \\<br>GAG AGG UUUGAACAGGGA UUU CUACAUAGAUUGU C<br>------- GC CUUU A AC AGUU
tmo-mir-1135	UUUA- GCA A - GUA<br>UACUCCCUCCGUUCGGAAUUACUUGUC GA AUGGAUGUAUCUAGA C \\<br>AUGAGGGAGGCAAGCCUUAAUGAACAG CU UACCUACAUAGAUCU G U<br>GUAAG AGC A U AUU
tmo-mir-1136	UG GU C UGUAU<br>UACUUCCUUCGUUC AAUUACUUGUCGCAG AUGGAUAUAU UAGA \\<br>AUGAGGGAGGCAAG UUAAUGAGCAGCGUC UACCUAUAUA AUCU U<br>GU UU A UAAUU
tmo-mir-1137	A G A - U<br>CCAAA UAAGUGUCUCAA CUU GUAC AACUUUG \\<br>GGUUU AUUCACAGAGUU GAA CAUG UUGAAAU A<br>C - A A C
tmo-mir-1432-5p	A A A G AU C--- A<br>GGGUCCUGUG UCAGG GAG UGACACCGAC CCG CGGAUGGGU GGCUU A<br>CUCGGGAUAC AGUCC CUC ACUGUGGUUG GGC GUCUGCCUA CCGGA C<br>A G C A CG CGUA C
tmo-mir-1436	CCUCAU G U AAC<br>GUACUCC UCCGUCCCAUAAUAUAAGAGCGUUUUU ACAC \\<br>CAUGAGG AGGCAGGGUAUUAUAUUCUUGCAAAAA UGUG A<br>------ G C AUC
tmo-mir-1584	-------- AGGAGAA AGAGGGAGAG AAGGGAA GGG<br>GCGGCGCU UG GAUC UC U<br>CGUCGUGG GC CUAG AG A<br>AGAGGAAG GGUAG-- GAA------- GGGGCAG AGG
tmo-mir-1878-3p	UC CA C U GC UG A U G<br>AAUCUUA AAC ACU UU AAACUUAGUCU GCACUAUAAAUUUA AAUG GCAUGU \\<br>UUAGAAU UUG UGA AA UUUGAGUUAGG UGUGAUGUUUAAAU UUAC CGUAUA A<br>-- C- C U UA CU A U A

S2 Table. (Continued).

tmo-mir-2120	A- G AGUAUAA A- UAGG A<br>CGU GAGGCCCAUCUGUCCCGGUU GAACCGGGACUAAAG UC GC U<br>GUA UUCCGGGUAGACAGGGCCAA CUUGGCCCUGAUUUC AG UG U<br>AA - CC----- CC CAA- A
tmo-mir-2538-5p	A CAGUGU UC- UC AUC AUUUUA UC<br>UUUGAGAGAG CCUUCG UUCA U CUCUAUU GU U<br>AGACUUUUUU GGAAGC AGGU A GAGAUAA CA U<br>- ACCCU- UUU UA A-- CGUACC CC
tmo-mir-2673	CC U UUCC------ CAG GCUGG U UG<br>UU UCUUCCUC UCGGCA GAC GUCGCCU AU C<br>AG GGGAGGAG AGCCGU UUG CGGCGGG UA C<br>GC C CACGCCACAU --- A---- U UC
tmo-mir-3348	CACA U - -- C- ---- .-GC AUU AG<br>AUAGC CC CCGCG AUCCU GCC GGGAG UCGCUGG CCA G<br>UGUCG GG GGCGU UAGGA CGG CCCUC AGCGACC GGU C<br>C--- U A AG AC AACC \ -- --- AC
tmo-mir-3630-3p	UC UG -- U U A- ---- AG ACA- G U<br>CA GAAACA AG GA GAU CCA AGAC GG UCA AACCC U<br>GU UUUUGU UC CU CUA GGU UCUG UC AGU UUGGG C<br>UA -- AG U - AG AAUA CA AGAC A G
tmo-mir-3682-5p	-UUU GA A -- UG --- AA---- UGUGAGAA<br>UG GU U AACC GU UGAGGGACU UGUGUU \<br>AC CA U UUGG UA ACUCCCUGA ACACAA U<br>--- AG G UU CU GUU AGAUGG UAGGAUGU
tmo-mir-3711	U UG U CGAGCCCUCCUUC C UCUCG- UCG<br>CGCGGG GC U G UAGCG CA GGU--CAGC C<br>GUGUCC UG G U GUCGC GU CCA GUCG C<br>- GU U U------------ U UAAAAA \ UCU
tmo-mir-4995	----- GA AA -U .-UU AA GAACG<br>UGG AGGGA G CAUAGGCAGUGGC GGUU GG G<br>GCC UCUCU U GUAUUCGUCACUG CCGA CC A<br>CGUGU AG AG - \ -- GG ACCCA
tmo-mir-5048	CUU UC UCUUGAC - C UUUUUG- ACC U<br>UGCUUU \ AGACC AAAAUC GUAA GC AUG U<br>ACGAAA U UCUGG UUUUGG CGUU CG UGC U<br>--- CU ------- A A UAUAUAA AUC U
tmo-mir-5049	G- AAA A C AACU<br>CUUUGAU GU UACUCCCUCC UCCCAAAAUAAGUGUCUCA \<br>GAGAUUA CA AUGAGGGAGG AGGGUUUUAUUCACAGAGU A<br>AG A-- - C CGAA
tmo-mir-5049-3p	C---------- AGU - UC .-G CAAUU<br>GACA AAU AUGGA GGAG GAGUAU U<br>UUGU UUA UACUU CCUU CUCGUA U<br>UGUCCAUGUAU CUU C UA \ - ACAUA
tmo-mir-5050	CGCCGUU GC- GA AGA C - A<br>UUGCUGGUUGAACGACCUCAUCAUG AC GC UCU CCU GGC C<br>AACGGCCAACUUGCUGGAGUGGUAC UG CG AGA GGA CUG U<br>------- AGC G- G-- A G U
tmo-mir-5054	UU- CU A AGCCAAAAACA -CUAACC - UUC<br>UGGA AU UGGA \ ACG UGGCCGUGGG \<br>ACUU UA ACCU A UGC GCUGGCACCC G<br>UUC UU A AAUUUUACAGU ------ G CUA
tmo-mir-5064	AAAUCC U UU U U AUUCAAU- UG U<br>UC GGUUGAA UGUCCAUAGCAUCA CCA CCUACC GG GC G<br>AG CCAACUU AUAGGUAUCGUGGU GGU GGAUGG CC CG A<br>GU---- C CG C - GUGGCAAC GU U

S2 Table. (Continued).

tmo-mir-5067	UC UC CG C C C A<br>GUACUCCUU GUU AUAUUAGUUGU CU AAA GGAUGUAUUUAG ACUU A<br>UAUGAGGGA CAA UAUAAUCAACA GA UUU CCUACAUAGAUC UGAA A<br>GU GA AA C A - U
tmo-mir-5073	UUGGU- UGAAUCGGAAA AU<br>UUGG CAAUUUU \<br>AACC GUUAAAA A<br>UUCUUU UUAAAAAUGGA AG
tmo-mir-5076	A .-AAUCAA AC AUC- UUCC C CGUG<br>GC UAGG \ CUCCC UUU AGGAAGAA A<br>CG AUCC U GAGGG AAA UCCUUUUU A<br>- \ ------ UU AGAC U--- U CUUA
tmo-mir-5079	UUCG- A .-U A UAG GA GUAC CACUUAU<br>CA UUUAU UAC UAG AACAAAUCCAAAUUG AGG GAAUUCUG \<br>GU AAAUA AUG GUU UUGUUUAGGUUUAAU UUC UUUAGGAU A<br>CUAAA A \ - - UUA A- ---- AGCUUCG
tmo-mir-5084	AAA- AC UCAUA U .-AUAGAU GU A CU AU<br>AAAAC \ GA UC GGUU GAUCCUCUGC GUA GU \<br>UUUUG U CU AG CCAA CUAGGAGAUG CAU CG G<br>CCAC GU UG--- U \ ------ UC A CG UA
tmo-mir-5141	AAUUAGA CUAU CG - UAUCCG CGCGUCG GU U AAAGC<br>CCAC UAA UUG GCU UCAGU G CUGCCGC CACUUG U<br>GGUG GUU AAC CGG GGUCA C GAUGGCG GUGAAC A<br>------- AAGC AU A UUAA-- AAA---- UU U AAAGU
tmo-mir-5174-5p	UUUCAU---- - CU UA -- C GAA A G<br>GC UUAAC AA AC UACUCCCUCUGU CCAUAAUAUAA CGUUUUUG CAUUA \<br>CG AGUUG UU UG AUGAGGGAGACA GGUAUUAUAUU GCAAAAAC GUGAU U<br>UAUUUUAUAC U U- UC CU A GUC G G
tmo-mir-5181	AACCAAUAAAUU A C A UG U AG<br>GUACUCC UC GAUCCA AAUAAGUGUCG GUUUUG ACUAAGGUU \<br>CAUGAGG AG CUAGGU UUAUUCACAGC CAAAAC UGAUUCUAA U<br>----------- G A A GU U CU
tmo-mir-5368	AAGA----- U GA A-- G UC A CG GG - GG A CA<br>GC CG UUCU ACCUU UG AG CC CG CCA AG AC GUCU \<br>CG GC GAGG UGGAA AC UC GG GC GGU UC UG CAGA G<br>CUUUGGAAA U G- CAA A CC C AU GG A UU A UG
tmo-mir-5387	A U AG<br>GCCCCC--CCUUUAGUACCG UUC \<br>CGGGGG GGAAAUCGUGGC AAG C<br>A \ C CA
tmo-mir-5523	AUGCAGA UAU U AUA UCC AUGUC UAU<br>UGA AAC AGUAA UGU UCCCC AACA U<br>ACU UUG UCAUU ACA AGGGG UUGU U<br>GAUAUAA U-- U C-- UUA GAU-- UAU
tmo-mir-6182	CAA CU ACA-------- UGU--- GG - A U<br>UGAC \ CGAG GUGAU A UGGCUUUG GCG \<br>AUUG A GCUC CACUA U ACUGGAAC CGC U<br>A-- AC AUCAAACAACG UUCUCC GU C C U
tmo-mir-6191	UCCA C G UGUCUAGAUA A- UA<br>UC GUCCCAAAUUACUU UCUUAGAUU UGGAUGUAUCU ACAC A<br>AG CAGGGUUUAAUGAA AGAAUCUAA ACCUACAUAGA UGUG A<br>UUAG A G ---------- CC CA
tmo-mir-6198	GAGA UUU - UU GUAUUGAC G U AUG U<br>ACC UC A CUCACCGA GGCUCU UCU GG GUCAUUC G<br>UGG AG U GGGUGGUU CCGAGG AGA CC UAGUGAG U<br>AAAG UCU U GG ACUUCAGU - - AA- U

S2 Table. (Continued).

tmo-mir-6203	AUCC .-CAA GAACAAUAAAAUUAA UU UU<br>AAGG AGGCGA AAU CUUC U<br>UUCC UCUGCU UUA GAGG U<br>---- \ --- AAUUCUUCUGGACG- GG CU
tmo-mir-6219-5p	U--- G CGUGUAA GAGA U<br>GC CCCUUUAGUCCCGGUU GAACCGGGACUAAAGGG GGUA U<br>CG GGGAAAUCAGGGCCAA CUUGGCCCUGAUUUCCC CCAU A<br>AUUC - AAG---- AG-- G
tmo-mir-6250	CCUG- ACU U----- GAUUCU U - UC<br>GAAAU CUU UGCGUU UCCUCUCUC UGA AAG UUGGCGGC C<br>CUUUG GAA GCGCAG AGGGGAGGG GCU UUC AACCGCCG C<br>CUAAA CU- CUAGAU ------ C U UA
tmo-mir-6478	A -- CGA AUCAAAA G- GGG<br>GC GCUGGGCUA GUUGGC GA UUCU \<br>UG UGACUCGAU CAGCCG CU AAGG A<br>- GU UC- AACA--- AA AGG
tmo-mir-6874-3p	A---- UU ACU CAUUUACCU---- UGC GAC<br>UAUA UUU GAGGGAAC AGUUC UGUUUU U<br>GUGU AAA UUCCCUUG UUAAG ACAAAG U<br>UUAAC UU CUU UUUAUUUUACUUU UU- ACC
tmo-mir-8155	AAAA -- C A .-G GG GU<br>CCUG GCU UG UACCACU UU GGAAU A<br>GGGC CGA AC GUGGUGG AA CUUUA G<br>GGGA AA U - \ - AA AU
tmo-mir-B6-3p	AC G CG AC CUCAACAA -- UUC<br>GGC GC GCU--GGUG \ CGUCUCC--GGCG CCGGG \<br>CCG UG CGA CCAC G GUAGAGG CCGC GGCCC C<br>-- - CU \ UC A-------- \ AG UGA
tmo-mir-l5-3p	A G CGA GGACG G -- - AA- CCU<br>CCAA GU A GCGUUGU GU UCUUC UC GUUG \<br>GGUU CA G CGUAGCA CA AGAAG AG CAGC G<br>- G AAA AGA-- G GA U GUG UCG
